# Older Adults and Family Perspective on Interaction with Nurses in Hospital: the Role of Mutual Understanding

**DOI:** 10.1093/geroni/igab046.3716

**Published:** 2021-12-17

**Authors:** Orly Tonkikh, Nurit Gur-Yaish, Ksenya Shulyaev, Amos Rogozinski, Elena Siegel

**Affiliations:** 1 University of California Davis, Sacramento, California, United States; 2 Oranim Academic College of Education, Haifa, HaZafon, Israel; 3 Faculty of Social Welfare and Health Science, University of Haifa, Haifa, Hefa, Israel; 4 University of Haifa, Nesher, HaZafon, Israel; 5 University of California, Davis, Sacramento, California, United States

## Abstract

Optimal nurse-patient-family interaction is required to provide effective family-centered care for hospitalized older adults and their families. This qualitative descriptive study explored nurses’ interactions with older adult patients and their family members during acute hospitalization. We used semi-structured interviews to collect data from a convenience sample of nine dyads of older adults (aged 62-85) and family members (7 children and 2 spouses) who accompanied them during an acute hospitalization in medical or surgical units. Interviews were performed via Zoom beginning in December 2020 until August 2021, 1-12 months after the hospitalization. Thematic analysis was used to inductively capture key patterns in data. Both patients and family members revealed three factors contributing to the way nurses interact with patients and families: (1) nurses’ recognition and understanding of patients’ needs for family members’ presence and participation in care; (2) nurses recognition that family members expect dedication of attention, beyond nurses’ focus on patient’s care (3) patient and family members’ recognition of the extreme workload of nursing staff. Participants described a range of informal approaches used by both nurses and families to address each other’s needs. Both patients and families emphasize the benefits and costs of nurses engaging in “exceptional” interactions with patients and families considering structural characteristics such as establishing a personal relationship or accepting family visits beyond the rules. The findings provide direction for further exploration of hospitalization structures and processes needed to support optimal nurses’ interactions with families accompanying older adults and family-centered approach training in acute care context.

